# "The Hospital" Nursing Mirror

**Published:** 1900-09-08

**Authors:** 


### "The Hospital" Nursing Mirror.

Being the Nursing Section of "The Hospital."

[Contributions for this Section of "The Hospital" should be addressed to the Editor, The Hospital, 28 & 29, Southampton Street, Strand, London, W.C., and should have the word "Nursing" plainly written in left-hand top corner of the envelope.]

### Notes on News from the Nursing World.

### NO. 9 GENERAL HOSPITAL, BLOEMFONTEIN.

A nursing sister, writing to us from the 9th General Hospital, Bloemfontein, on July 27th, condemns the allegation about the mismanagement of the hospitals. She says: "No doubt there is a spice of truth in them so far as the field hospitals are concerned, but everything was done that could be done at the time. When we first came into Bloemfontein no medical comforts nor stores could be brought up the line, and field hospitals are not supposed to have bedsteads. . . . It seems hard lines to be abused . . . when everyone has done their level best. . . . One hospital is really a wonder. It was equipped for 500 patients, and it had to be expanded to hold 1,700, and the same staff to work it. The poor fellows were all well looked after, and every one of them is indignant that such reports should have been spread." Our correspondent acknowledges, however, that the sisters have had "a very rough time of it altogether," and that on a wet day "there is not a dry rag inside or out." She adds that she feels very well and never gets cold, and that "it is glorious living always in the open air."

### MACKENZIE'S FARM BRANCH OF THE IMPERIAL YEOMANRY HOSPITAL.

The new P.M.O. of Mackenzie's Farm branch of the Imperial Yeomanry Hospital, Mr. W. Turner, reports that Sister Stevenson has been appointed lady superintendent, and Sisters Cable and Cooper nursing sisters. The sisters' hut, consisting of six rooms, including kitchen, was opened on the 6th of last month. This hospital is now entirely under the control of the Imperial Yeomanry Hospital Committee. The ward holds 25 patients, and was originally merely a detention ward; but since the sick and wounded were admitted the staff have been working all day and half the night.

### CHANGES IN MILITARY HOSPITALS.

A large increase of nursing sisters has recently taken place in the Southern Military District, Portsmouth being the head-quarters. At the station hospital there, the staff numbers ten sisters, exclusive of the superintendent; Gosport has eight, and the smaller hospitals of Hillsea, Parkhurst (Isle of Wight), Winchester, Dorchester, and Devizes, have all two sisters working in each. Before the war the usual number of sisters for the district was six only. The whole of the new arrivals belong to the Army Nursing Service Reserve, and consist of the following: Sister Edwards, stationed at Hillsea; Sisters Morrison, Lawrence, Luscombe, Sanders, Neville, Steenson, Gilbert, Bentharn, and Biggs, stationed at Portsmouth; Sisters Moffat and Wetton, stationed at Winchester; Sisters Hinton and Schliemann, stationed at Devizes; Sisters Read and Breen, stationed at Parkhurst.

### THE BOER PRISONERS AND MARY KINGSLEY.

"On Saturday, August 11th," writes a nurse at Cape Town, "the prisoners, who are at present confined in Green Point Camp, Cape Town, gave an entertainment the proceeds of which were to be given to the Mary Kingsley Memorial Fund. The concert was a great success, and an excellent programme was provided, consisting of Christy Minstrel and other songs; the duet 'We Shall Never Be Happy Again' was no doubt chosen to suit the feelings of some of the prisoners, who, nevertheless, looked happy enough. The result of the entertainment was that the sum of £10 was handed to the treasurer of the fund, a touching tribute to the memory of one who was so much loved and respected."

### AN AUSTRALASIAN TRAINED NURSES' ASSOCIATION.

The first annual meeting of the Australasian Trained Nurses' Association was held in the Royal Society's Rooms, Sydney, on July 6th. Established last year under the title of the New South Wales Trained Nurses' Association, the scheme has progressed so steadily, and met with such encouragement from nurses and medical men, not only in New South Wales but in the other colonies, that the council have widened its bounds and altered its designation to embrace the whole of the Australian Continent. The rules have been modified and amended for the better in one or two directions, notably in the extension of the time during which nurses who may be unable to produce a certificate of training, but who can show evidence of good work and competency, will be admitted to the privileges of the association at the discretion of the council. On this body are a number of the most prominent medical men in Sydney. Miss McGahey, matron of the Prince Alfred Hospital, in whose hands their interests will confidently be left by Australian nurses, is one of the hon. secretaries; and matrons, sisters, and nurses are well and adequately represented. The association now numbers 388 members, has issued its register, and is fairly launched on its career, which it is cordially hoped may be a prosperous and useful one. The report, of which we have received a copy, states that the council proposes during the next year to bring the association within touch of the other colonies by the formation of small local executive committees, to form auxiliary branches for midwifery and mental nurses, and to initiate a benevolent fund scheme for the benefit of nurses in need of temporary assistance. As constituted the association bids fair to become, in the words of the council, "of great benefit to the nurses themselves, to the hospitals throughout Australasia, and to the public in general."

### UNIVERSITY HOSPITAL EXTENSION.

The new wing for the accommodation of the resident officers of University College Hospital will not be ready for several months yet, although the hospital itself will be opened for patients this month. At present only the matron and two or three of the sisters live in the hospital buildings. When the new wing is completed there will be accommodation for about 150 nurses, who are now residing in rooms in the neighbourhood. While the alterations have been proceeding the only department not closed has been the maternity department; this has been kept open as usual.

### THE NURSES OF MANCHESTER INFIRMARY.

The charges made in *Truth* against the Board of Management of the Manchester Royal Infirmary have been met in a prompt and straightforward manner by the chairman and his colleagues. With reference to the complaint of a nurse that the lady superintendent declined to give her a "testimonial," the answer is that the nurse in question failed to pass both the medical and surgical examination, and that "it is not the practice to give testimonials which might be considered as equivalent to certificates." The lady superintendent is, however, always ready to reply to inquiries as to the character and competence of any nurse who has been trained in the institution. Nothing could be more fair both to the public and to the nurses than this. The nurse asserted that during the three years she had been in the infirmary an undue proportion of her time was spent in the surgical wards, but the Board point out that a much larger proportion of the patients are surgical cases, that the experience obtained in the surgical wards is considered the more valuable and is the more sought after. She also complains with respect to the examination, at which she failed, that she and three other nurses had not only been on duty all the previous night, "but had been called up the previous day four hours before the usual time in order to be put through some sort of rehearsal by the matron." The Board frankly admit that the calling up of the night nurses so much earlier than usual was owing to a mistake, and "is much to be regretted." So it is, and such mistakes should be avoided in future. We also think that the custom of "examining nurses on the morning following night duty," which the Board only defend on the ground that objection has not hitherto been raised, should be discontinued.

### THE DERRY GUARDIANS AND THEIR NURSES.

A lady member of the Derry Board of Guardians has attacked some of the other members of that body because they only offered a salary of £35 a-year, "with apartments and rations," to a properly-qualified trained assistant night nurse. It is fair to point out that in the centre of England the remuneration offered is on much the same scale. The West Bromwich Board of Guardians have just decided to appoint two night nurses, "non-resident," at a salary of £1 5s. per week. Moreover, Mrs. Morris, who made the attack in question, and subsequently moved that "a head nurse be appointed," was met by a reply that a head nurse had already been appointed by the Local Government Board. Boards of Guardians have sins enough to answer for without accusing them of misdeeds which they do not commit; and assuming, of course, that the head nurse at the Derry Infirmary is fully trained, the addition of an assistant night nurse on the terms mentioned seems to meet the necessities of the case. We are glad to see that the Derry Guardians have sanctioned the expenditure requisite for the provision of nurses' rooms in the new infirmary buildings.

### A SCANDAL AT SHEPTON MALLET.

We have received the following letter from Nurse Hobson, of Shepton Mallet, which we think is important in several ways. First, it shows into what a sad condition a workhouse infirmary can drift in the absence of proper care and the constant supervision of a nurse trained up to the hospital standard. Next, it proves that the way to deal with such problems is to go straight to the fountain-head—the Board of Guardians, and put the matter plainly before them. There are often curious specimens on Boards of Guardians, but in every board many, and in some quite a large majority of the guardians are men who are thoroughly desirous to deal well and kindly with those under their care, and if an abuse is proved to them they will try to rectify it. We imagine from the latter part of Nurse Hobson's letter that this has been the case at Shepton Mallet, and we hope that the result of her laying the matter before the board will be that a great improvement in the condition of the infirmary will take place.

"With reference to the paragraph reported in your issue on August 18th, *re* a 'Scandal at Shepton Mallet,' I beg to say my reason for saying I was afraid of having typhoid myself was that there were already three cases in the house, which I found getting in and out of bed as best they could with temperatures 104, 103·8, and 103. Two of them had hæmorrhage, and a fourth case of typhoid developed after I came. In the female side of the infirmary all the w.c.'s were blocked up, and a sink had been covered over with boards and nailed down to prevent the inmates throwing rubbish down, the master told me. However, a hole about two and a-half inches had been left for the water from the tap to drop, and down this hole had been thrown into the sink old tea leaves, bits of food, &c., and worms had developed there. In another ward I found three children with measles—one two years, another three years, and a boy of ten, who told me that he had not been washed for a fortnight. I asked a girl of 19 years, who had no experience of nursing, but had been appointed as assistant nurse about four months ago, if it was a fact that these children had not been washed, and she said, 'Yes; the nurse who left told me not to wash children with measles, and we have had ten or twelve cases.' The two younger children's beds were soaked with urine, while on the male side of the infirmary there was nearly half a bath of old linen left to soak that a man had taken off his ulcerated leg, which he had intended to wash, he told me, but felt too ill to do so. This soiled linen had become quite putrid, and the other patients had to wash from the tap over them, as they had no other convenience. I told this man how very dirty I considered such a state of affairs. He smiled, and said, 'Why, nurse, you don't know what dirt is.' I was given a mattress to sleep on worse than the inmates have, with vermin in it. It has been condemned now and taken away, and the state of the ward beds were as reported in the *Bristol Times*. I called the attention of the matron to all these things. I have been maligned and accused of exaggerating since the filth has been taken away, but I repeat that it was almost impossible to exaggerate. Now for the brighter side of things. I have been given a temporary 'trained nurse' to help me, and also a paid scrubber; two strong boys come up each morning to scrub in the male infirmary. The drains have been attended to, and although it has been hard work, I have the satisfaction of knowing that I have tried to do my duty."

### SHORT ITEMS.

Miss Julia Riley, who has been charge nurse at one of the infirmaries under the jurisdiction of the Eccleshall Board of Guardians for about two and a-half years, has resigned her appointment, in order to be trained as midwife to qualify herself for a higher post.

### Lectures on Medicine to Nurses.

By H. A. Latimer, M.D. (Dunelm), M.R.C.S. Eng., L.S.A.Lond.; Consulting Surgeon, Swansea Hospital; Past President of the South Wales and Monmouthshire Branch of Brit. Med. Assoc.; Past President of the Swansea Medical Society; Hon. Life Member of the St. John Ambulance Association, &c.

### SIGNS—FUNCTIONS OF THE SKIN—RASHES—SOURCE OF BODY-HEAT.

The closing part of my last lecture was devoted to an explanation of the meaning of the words "symptom" and "sign," and you will remember that I differentiated between those terms. I would not have you think that the only signs we meet with in disease are connected with skin eruptions; as a matter of fact, many diseases are marked as to their personality by characteristic phenomena, the appearance of which on the scene stamp their individuality. As one or two examples of this truth I would cite the tremors of St. Vitus dance, and the short-lived and characteristic convulsions of epilepsy. But, undoubtedly, the most marked illnesses which have their identity stamped by signs are those fevers accompanied by rashes, for, in the truest sense of the word, such rashes are "pathognomonic," a word which you will often hear used with respect to them, and which, being derived from two Greek ones meaning "to know suffering," is applied to a peculiar characteristic sign of a disease. For if with fever and catarrhal symptoms the peculiar, papular, crescentic-shaped rash of measles makes its appearance, the diagnosis of that disease is clenched; if with fever, diarrhœa, and chest dulness, peculiar rose-coloured spots make their appearance on the body, in the second week of the illness, such spots are pathognomonic of enteric fever. I could multiply such examples bearing on this subject, but as I feel that I have said enough on the point I will pass on to other matters.

It is well to note the life history of skin eruptions. Each exanthem has its own peculiarity in respect to its mode of appearance, character, duration, and decline. The peculiar appearance of the skin is undoubtedly due to an inflammation there; an excess of blood presents itself in one or more of its layers, and, as is usual in inflammatory affections, some of the watery parts of the blood and some of its cells escape from the vessels and lie beneath or between the meshes of the epidermis. Skin is not the simple membrane which persons unskilled in anatomy might deem it to be. When examined under the microscope it is found to be made up of two distinct layers, which are respectively called the epidermis, or cuticle, and the derma, or true skin. These layers are again sub-divided in a manner I need hardly dwell upon, as it is undesirable to burden your minds with refinements of this sort. It is desirable, however, that you should remember that an abundant supply of nerves and blood-vessels exist in the skin, and that its continuity is broken by sweat glands and ducts, hairs, and by the minute ducts, or channels, conveying an unctuous secretion to the surface, for the purpose of rendering it supple and soft. A few references to facts of every day occurrence will also show you what important functions are fulfilled by the skin, and will serve to show you how easily it is affected by influences from without and from within the body.

Let us, first of all, consider the changes wrought in its blood supply through the intervention of the nervous system. See how an emotion of pleasure or of shyness will cause a person to blush; see how an emotion of fear will cause a person to become pallid and blanched; in these cases the excess of redness on the one hand, and the absence of colour on the other, are due to actual changes of size in the minute blood vessels which are distributed to the skin, through their dilatation or contraction in response to currents conveyed by sympathetic nerve fibres which are supplied to them. Look at another common phenomenon—the so-called "goose-skin" contraction caused by cold or by the hearing of some ghost story which has made you feel "creepy." Here the muscular fibres which lie in the deeper layer of the skin have contracted in response to stimulation, and have puckered the membrane into minute points.

As nurses, you see medicines introduced into the system by means of hypodermic injections or by inunction with ointments, and you have doubtless observed the extreme rapidity with which they exert their influence conveyed by this route, and the smallness of the doses which it is necessary to use to produce definite results. Has it ever occurred to you to reflect upon the mechanism which has been at work on these occasions? Here, at any rate, is the explanation of the subject: Lymphatic, or absorbent, vessels are present in the skin and beneath it, as elsewhere in the body, and the drugs which are placed there are rapidly absorbed by these vessels and are quickly conveyed into the blood to affect remote parts of the general organism.

These remarks of mine are introduced here to show what a variety of structure lies in this delicate part we call the skin. It serves to explain, in a certain measure, the varieties and diversities of rashes, or it shows, at least, how it is possible by muscular and vascular derangements there to produce inflammations of various types and arrangements. Why each morbid influence should produce its peculiar and characteristic exanthem is another affair. I presume it is in response to design, just as each flower, plant, tree, or animal has its peculiarities. When the occasion arises during a description of any disease accompanied by a rash I shall describe in some detail the character of the same, and shall deal with the various points of interest which occur in connection with it. I need say little more at present than that, as nurses, it behoves you to pay particular attention to certain matters touching on the general question of eruptions, for you can give great assistance to the physician by reporting to him when the same first appeared, on what part of the body it first showed itself, and in what sequence it appeared elsewhere; how long it remained in each affected part, how it disappeared, and what phenomena accompanied its coming or going. These details ought to be easily borne in your minds, and I assure you they can be made great use of in helping the attendant to form a diagnosis or to give a prognosis. I am at present dealing, in a general manner, with the subject of the acute fevers, and this would seem to be an appropriate occasion to describe the fever-process and to explain the mechanism of the production of body-heat and the physiological laws which prevail in controlling the same. It is an interesting fact to be noted in the first place that, given a condition of health, the temperature of our bodies is maintained at a mean average of 98·4 deg. Fahrenheit in spite of the varieties of climates in which men live. It does not actually stay at this point, for careful observations show that there is a daily rise and fall in body-heat, the temperature rising slightly from eight a.m. to the afternoon, and falling again from eight p.m. until the small hours of the morning. This rise and fall—the diurnal range, as it is called—is greater in youth than in manhood, and may then vary from between one and a-half to two degrees in extent, falling to 97·5 deg. in one direction and rising to 99·5 deg. in another; any persistent rise above the latter height marks fever. The first question to be answered is, What is the source of heat in the body? Knowledge of physiology tells us plainly that this warmth is derived from the combustion of foods and tissues within us much as it is evolved by a burning fire in a grate in one of our houses.

### Experiences in a Plague Hospital.

By a Nursing Sister.

A few details of the working and management of a native plague hospital, and the efforts made to arrest its deadly ravages in India will be read with special interest just now. Neither expense, trouble, or talent is being spared to fight this terrible foe, but in spite of all efforts the mortality in the city of Bombay has been higher than ever. It is a little discouraging to us who are working so hard, and we are inclined to wonder if we are of any use here till we turn with pride and satisfaction to the large ward full of convalescents, many of whom we know we have snatched from the jaws of death, and then we feel comforted and return more hopefully to those still in the acute stage, whose recovery seems well-nigh impossible.

### Inoculation.

I have been nursing during this last epidemic in a small hospital in Bombay. Soon after our arrival we were all inoculated at Parel; the doctors were done first, and then the nurses. One sleeve was rolled up, and the operator went from one to another with a big syringe, and gave us the dose. It did not hurt, nor was I ill afterwards, but most of the others seem to have been bad. My arm was swollen and rather painful for a short time, but my general health was not affected. A week later I was operated on the other arm, and after the second inoculation I was very poorly and had a temperature of nearly 103; but I was all right again the following day and able to take a drive.

### The Bungalow.

We are obliged to wear topees, or sun hats, to walk across the compound from our bungalow to the wards. The bungalow in which we sisters live consists of three rooms and a verandah where we take our meals; it is built of wood, just rough planks nailed one over the other, and the roof is covered with matting. Each room has a very primitive bathroom behind it. The insects are terrible, and our dinner table is covered with jumping and flying beasts; we dislike the grasshoppers most, they come so suddenly and are so terribly large.

### The Hospital.

This is only a temporary hospital, run up about three years ago. One ward has just been pulled down and replaced by another of wood with an iron roof. Our bungalow has also been enlarged and improved since my arrival. The old wards are made of bamboo matting with plenty of windows and ventilation. The roofs are of tarpaulin, supported by bamboo poles, which are very much in the way, as there are so many of them. One roof is thatched with palm leaves, and a regiment of sparrows have taken up their abode in it, building their nests, rearing families, and behaving as if there were no human beings within a mile of them. London sparrows are tame enough, but these are positively impertinent. The floors are merely the rough earth, banked up a few inches from the ground outside; they are very uneven, which is most tiring. Once a week, and oftener if necessary, lime is freely sprinkled over them—a most disagreeable performance, as for hours after it is flying about down our throats, into our eyes and hair, and spoiling shoes and skirts. Several times during the day the floors are watered with lotion, and brushed all over by the sweepers with twig besoms.

### The Patients.

Although ours is supposed to be a "plague" hospital, quite half the patients are "observation" cases suffering from pneumonia, fevers, and sometimes phthisis. A case is rarely refused, though many ought by rights to be in a general hospital. A great many are purely starvation cases, often picked up on the roadside, having come from a famine district. These patients are just skin and bone, and nearly always die, as they are too far gone for food and medicine to have any effect upon them.

### The Examination.

All patients as they come in are examined by the hospital assistant, as they lie on the ambulance, to see if they are plague cases or not; and he directs to which ward they are to be taken. They are at once put to bed and washed by the ward servants, and unless they are very bad the sister is not allowed to look at them till they are quite clean, arrayed in nice flannelette jackets, and covered with comfortable red blankets. Then they are ready for a thorough examination; particulars of cases are written out, medicine and treatment ordered, and the sister enters each patient in the ward-book by the number of his cot. Many of them have never slept in a bed before, and don't like their jackets, which they take off and use as a pillow. Feeders are a deep mystery to them; some learn to behave nicely, and become orderly, pleasant patients; but others keep their jungle habits, and are a great trial to their nurses. As a rule, a relation or friend stays with them, and is often of the greatest use in feeding and generally looking after the patient. Sometimes they give a great deal of trouble, objecting to the treatment, &c., and then they are turned out of the ward unless they promise to behave better.

### A Mem Sahib.

The sisters now have a very different time from those who came out first. The natives are no longer afraid of us, and take it quite naturally that a "Mem Sahib" should feed and nurse them. The children are as friendly as possible, and soon get devoted to their "Sister Sahib." I remember one man being very much surprised when I went up to him to take his temperature, and he did not quite like it; but the ward boy hastened to assure him that "Miss Sahib" was a "bote utcha ayah" (a very good nurse). We were all much amused, "ayah" being the only equivalent in Hindustani for "nurse."

### The Castes.

We have a great deal of trouble, of course, with the different castes, and every day one seems to learn about some new fad. The high caste patients, as a rule, do not come to us, but go to their own special hospital. Nearly every caste has its own little hospital, under native treatment and management, to which they go if they like. We had a Brahmin once who insisted on the Brahmin cook coming to bring him his food; he would take it from no one else. Another one had milk brought in by his brother at first, but presently consented to take the hospital milk if it was given him by a Brahmin; and as his friend used to leave him at night, I had to wake up the relation of another patient to feed him. The old fellow was very good about it, and fed him for several nights.

### The Dinner Hour.

Dinner-time in the wards is rather amusing. The two cooks come in with a huge charger of rice, and pots of meat, vegetable, and a liquid looking like soup, all of which are put on the ground. Those who are well enough bring up their own plates and squat round the cook, waiting to be served. He doles out the rice with his hand and the other things are ladled out with a spoon. As soon as a man is helped he retires to his bedside, and sits on the ground before such a piled-up plate as would take a European a week to get through. They eat entirely with their fingers, and manage to convey the rice to their mouths in the tidiest manner. The dinner is at eleven a.m., and at six p.m. they have supper, the same sort of meal, only not so much of it, and they have "chupattis" as well. "Chupattis" are their bread, and look like a large pancake; they are very nice when light and freshly made.

(*To be continued.*)

### Nursing on the "Orotava."

### A CHAT WITH ONE OF THE SISTERS IN CHARGE.

By a Correspondent.

The "Orotava" had a very rough experience to begin with, though later her passage was smoother and very pleasant. Even my informant, who is an excellent sailor, felt the effects of the rolling and pitching.

"It was not a graceful roll," she said, laughing, "it was a sudden lurch, and she went down into the trough of the sea until you wondered when she was coming up again."

"Had you a long voyage?" I asked.

"No; we left Durban on the 4th, and arrived on the 27th."

"How many sisters were on board?"

"Four—Sisters Allen, Mellor, Mary of the Sacred Heart, and Stewart."

"And who was in charge?"

"There was no question of anyone being in charge; we shared the duty."

"Where did the patients mostly come from?"

"Some came from Volksrust, and others from Mooi River Hospital."

### Humours of the Voyage.

"Oh, I must tell you what happened to me one day when I was in the pantry. I was preparing an egg fillip, and the steward had given me a mat to stand on, telling me I should get my feet wet. The floor of the pantry—a long room—is tiled; the ship plunged, and I slid the length of the room. It was a very narrow escape of a broken leg, for I caught my foot under one of the foot warmers which stood in a row along the walls."

And then the Sister, who has the gift of the comic spirit, drew a picture—in words—of a moment, when a sudden rush of water, coming through the open portholes, almost drowned two of the nurses who happened to be passing at the moment:—

"'Oh, Goodness!' one exclaimed, 'where am I?'

"They looked just like drowned rats—clothes hanging down, heavy with sea water, and with seaweed clinging to them. We all had a great deal of fun on board, I assure you; the Tommies are so amusing, especially the Irish ones.

"'Begorrah, Sister,' one of them said one day, 'the devil's got hold of my stomach!'

"'What do you mean?' I asked.

"'He has, Sister; he's got hold of it properly.'

"Then they are so funny about being ill. Often a man will come to you and complain of a pain.

"'Where is the pain?' you ask.

"'In my chest, Sister.'

"'And where does it go then?'

"'Up into my ears, Sister. I think some stout would do me good.'

"Then you send him to bed, and put him on milk diet. He doesn't like the milk diet, but he puts up with it for the sake of the stout."

### Not a Hospital Ship.

"Is the 'Orotava' a hospital ship?" I inquired.

"No, she is a transport. Consequently, we had to transfer one of our patients who was really very ill before we left the Cape. He hated leaving us. 'I don't want to go, Sister,' he said; 'I'm sure I shall soon get well if I stay with you.' But we were not supposed to take serious cases, so he had to go on a hospital ship. You see we had no convenience for operating if anything happened during the voyage, so we could only bring convalescents."

"Were they mostly fever cases?"

"Yes, and dysentery and pneumonia. There were a few surgical cases, but all were getting better."

"How many were there on board?"

"I should think quite 800 altogether. But only six of these came to the hospital."

"Then there was not much to do?"

"No, very little, except at the beginning of the voyage, when nearly everyone was sea-sick. Would you like to hear what is provided for invalid Tommy on board ship?"

"Very much."

### Tommy's Meals.

"I wish I had a menu to show you, but I think I can remember. For breakfast they have bread and butter, tea or coffee, Irish stew and molasses. For dinner, roast beef and vegetables, and any who are ordered by the doctors have stout during the morning. Then for tea they have stewed fruit and tea or coffee. The Government looks after its soldiers well. There is no stint of food nor of stimulants—whatever the doctors order is given generously.

### The Medical Staff.

"I can't speak too highly of our medical officers," the Sister went on. "They were simply devoted to the men, and would do anything for them. Dr. Rawton, Dr. Parker, and Major Doman, the medical officer in charge, were all exceedingly kind, both to the patients and to the nurses. We had a great many things from the A. B. Fund, and the doctors were so kind in giving them out. All we had to do was to ask the name and regiment of the soldier who needed help, and the clothes were given at once. We were obliged to be particular in our inquiries, for Tommy always gets more if he can!"

"Had the men lost many things?"

"Yes, one man came and said he had lost his overcoat, and when we had inquired his name and regiment he was compensated for all he had lost. Just as we were arriving on the last morning the medical officer came to me and said, 'Sister, we have still some things left; do see whether anyone wants anything.' But they were too excited at the prospect of their arrival home to want anything then.

"They were so good in getting up entertainments too," the Sister went on, "we had three concerts during the voyage and a fancy dress ball."

### The Devotion of the Nuns.

In the course of our chat I heard several stories of the devotion of the nuns during the war.

"I know for a certainty," said the Sister, "of more than one nun dying of starvation because she had given up her food to the soldiers. At Estcourt it was so. The Superior herself died of starvation, though it was supposed to be enteric fever. Sister Mary of the Sacred Heart, who came over with us in the 'Orotava,' has been five years in South Africa, and is going to Canada. She has been many years a nurse."

### Busybodies Again.

"Have you been troubled by the 'busybodies?'"

"I will give you an instance of the harm they do. You know there's not a Tommy who hasn't a chum, and when, for example, a lady visitor gives fruit to a surgical patient he is pretty sure to share it, and for all you know he will give it to a man with enteric fever. Isn't that bad enough? I know of an officer at Maritzburg who absolutely refuses to have visitors in the hospital; he would rather they went to Hong Kong. And he doesn't mind telling them so. Then, the complaint has been made that milk was given to fever patients and to men suffering from other complaints out of the same vessel. It was no such thing. I will tell you what we did. We knocked down the edges of the condensed milk tins—they make very good cups—and marked them 'For fever,' 'For dysentery,' &c. We never used the same vessels for all alike."

### Nurses at Norval's Pont Stationary Hospital, Cape Colony.

BY A CORRESPONDENT.

"FOUR sisters wanted for Norval's Pont." "Where is Norval's Pont?" and "Shall I be chosen?" were the questions we all asked ourselves at Wynberg. "Oh! Norval's Pont is at the other end of nowhere." "No one seems to know anything about it." "It is on the borders of the Free State. The Boers destroyed the bridge, and a suspension affair was started. I was offered a ride in the basket." "Oh, yes! Then they had a pontoon bridge, and now the old bridge has been rebuilt; there is a convalescent hospital there." "Why do they want sisters for a convalescent hospital." Nobody knows. "Please miss, Sister Garrioch says will you pack at once and hold yourself in readiness to start for Norval's Pont to-night." I and three others packed and held ourselves in readiness for a week in varying stages of uncomfortableness, according to the amount of unpacking subsequently done. At last Sunday came round again, and we had orders to proceed by the night mail to Bloemfontein. Our boxes were labelled and on the cart when our orders were again changed to Norval's Pont, and what a relabelling there was at the station. Crowds of friends to see us off, good-byes all round, and while some of us were still on the platform the whistle went, and before we could get into the train she had begun to move. Then there was a settling down, a wondering how four of us, with all our small luggage, would pack into one compartment, and live there for thirty-six hours. Fortunately, the weather was cool, even cold, and we were glad of the hot-water tins supplied at Cape Town, and once again later in the night. There were several doctors in the same carriage going to Bloemfontein, and they besieged our compartment from time to time for tea and cheerful conversation. One of us had brought a pack of cards, which helped to while away the time. One day and two nights in the train, and we reached Norval's Pont at about nine a.m.—a sort of basin with kopjes all round, a river, a little village, a few trees, and a camp. The bridge has been rebuilt, and is now in use again, though the pontoon and temporary railway bridges are still in existence.

We were met by Colonel Falney, who made us honorary members of the mess for the day, till we should have started our own. Very glad we were of breakfast, bacon, eggs, and hot coffee; it was refreshing after thirty-six hours in the train, where we mostly provided our own meals.

### THE EDINBURGH HOSPITAL.

After breakfast we were taken to see the two marquees in course of preparation for our reception, and then away we went to call on Miss Gill, lady superintendent at the Edinburgh Hospital close by. She very kindly showed us over. It consists of one-storied iron buildings, lined with green canvas, cool and refreshing to the eye. They seem to have every appliance and convenience which can be wished for, and the patients appear to be as comfortable as in any old-established hospital at home. Returning to the camp we met at lunch Lady Gifford and her sister, then guests of the colonel, who were starting a convalescent branch for officers. In the afternoon we were told that we should probably have a few days' grace in which to settle down and get things into working order before any patients were sent up. Hitherto serious cases, or those likely to become so, had been passed on to the Edinburgh or to other hospitals on the lines of communication, as there had been no sisters here.

### OUR QUARTERS.

We were given for ourselves two marquees, each about 27 ft. by 10 ft., with boarded floors and double canvas, one as a bed and the other as a sitting-room. Army regulation tables on substantial iron trestles were supplied for both marquees, and comfortable iron spring beds—with mattresses in sections, apparently for the convenience of packing—nice white sheets and counterpanes, with regulation brown blankets. Everything is marked with a brown arrow and a number, so that we felt like prisoners. Our marquees have windows in the roof for ventilation and for light, if all the openings—of which there are four to each marquee—have to be closed, as is pretty often the case on account of wind or cold.

Our kitchen arrangements were very simple at first; two rails on bricks laid on the veldt and four pots. A water barrel on wheels was added shortly, our water supply being renewed from time to time with the assistance of four mules and two natives from a distance of some miles away. A little later a field oven was added, a very primitive and more or less efficacious variation of the genuine thing, made in a rough and ready way with materials to be found on the spot. A cook and an orderly were forthcoming at once, and later we acquired a cook's and an orderly's assistant.

### A DEADLOCK IN PATIENTS.

It is now close on three weeks since we arrived, and there seems to be a deadlock in patients. The camp developed six enterics for our benefit immediately on our arrival, but our excellent nursing seems to have stemmed the tide of disease, and those six patients are all doing well; several of them are already almost convalescent, and we are wondering if we shall be moved on elsewhere, as our services at present do not appear to be much needed here. There is little work and no excitement beyond meeting the mail trains or waving to them as they pass. In the evening we get the *Bloemfontein Post*, a paper of four pages, containing, as a rule, very little news of the outside world. We live, however, in daily anticipation of mail letters; they straggle in at all times, it may be that they are dated several mails back, but that is nothing. These errant letters have been to Bloemfontein or to Pretoria, or perchance taken an excursion into Natal by way of a change, and when they do turn up are doubly valuable; valuable for the post marks they sport, as well as for the belated news they contain of friends far away, who fondly imagine that they are keeping us regularly posted up in all home news.

### PLEASANT DIVERSIONS.

Ours is not the only camp here. On the other side of the hills is a detachment of Nisbet's Horse, and then there is the Engineers' camp not far distant. The men ought to have a good time. There is plenty of buck shooting in the neighbourhood, and an occasional haunch or shoulder of venison makes a pleasant change from the beef, mutton, and bully beef of rations. Then, too, the horns are pretty, and can be taken home as trophies. There are several Dutch farms about, and there is always possible the excitement of being chased by an ostrich when one takes one's walks abroad. Also you can go to the store and invest in such delicacies as potted meat and pickles to vary the monotonies of afternoon tea, which, after the manner of hospitals at home, seems to be rather a favoured meal here. Seats in our marquee for that repast are usually at a premium, and we shall soon have to request our visitors to bring their own. Yes, life at Norval's Pont has its amenities.

### Wants and Workers.

SISTER KATIE writes: I wonder if any nurses are able and willing to help a fellow nurse to earn her living by needlework. Owing to a severe internal operation, she is unable to return to her profession, and thinks that she may perhaps help herself and other nurses by doing any sort of mending, stocking darning, making aprons, or underclothes. She is a very good plain needlewoman, and hopes in a short time to get sufficient orders to enable her to buy a machine. She will willingly pay postage one way, and will undertake that the work shall be thoroughly well done and quickly returned.

### Some Hospitals in the Netherlands-Indies.

### II.—THE BERI BERI HOSPITAL, BUITENZORG.

By a Nurse on her Holiday.

To anybody interested in tropical agriculture the nurseries and laboratories established at the Langbau afford a treat. Their object is to enable the staff to watch the growth of, and determine the most favourable methods of cultivating, the various useful plants and trees of warm countries, and to disseminate among planters and agriculturists the information thus gained. The gardens cover 180 acres of ground, and, besides annually publishing the results of its investigations, the department gives away seeds, cuttings, and young plants to any *bonâ fide* residents of the Dutch East Indies who apply for them.

There is a pharmacological section where drugs are grown for experimentation in the laboratory attached to the old garden, and there are special divisions for the study and improvement of Sumatra tobacco, and of coffee culture. Here, too, is the laboratory of agricultural chemistry, built only nine years ago, and equipped in a quite up-to-date manner.

Attention is given to the best modes of growing the numerous varieties of rice met with in the East, some of which, when consumed as a staple diet, seem to possess a marked influence in the etiology of beri beri—or at least some form of peripheral polyneuritis, as the specialists term it—while other kinds, chiefly by the medium of their nitrogenous cortex, appear to offer a remedial food against the same disease. The methods of terracing, puddling, and irrigating the *sawa* lands, or padi fields, lend quite a special feature to the landscape, and are not less interesting to the mind than charming to the view.

Here, too, we see several kinds of tea, of sugar-cane, of cacao, of gutta-percha, and of spices, besides drugs. It is pleasing to meet with the *Erythroxylon coca* in its native state, from which the cocain of our eye wards and out-patient rooms is derived; the sarsaparilla vine, too, the vanilla creeper, the annatto shrub, the ebony tree, the white-flowered variety of pomegranate bush, the pretty logwood, the malodorous cinnamon, and a host of others, of which not the least valuable and interesting is the rare *Strophanthus dichotomus*, from whose seeds a powerful cardiac stimulant, now obtainable in the drug market, is prepared. I lingered between the beds of plants until the sun was high in the heavens, and then bent my steps towards the inn, intending to visit, later in the day, the institution known as the "Ruma Sakit Beri beri"—or beri beri hospital—one of my chief objects of assault in Buitenzorg.

### The Buildings.

Fortified by tiffin, and armed with a letter of introduction from headquarters to the Medical Superintendent, I somewhat timidly telephoned my name and wishes to that gentleman, and asked for an appointment. The doctor was more than polite, for he bid me stay where I was, and in ten minutes he would come round in his carriage and would drive me to the hospital himself. We spoke chiefly in French, but he often broke out into whole sentences of Dutch and I into words here and there of English, with a little Platt-Deutsch thrown in—a dialect I had picked up in Hanover after my school days. We soon arrived at the Beri beri Hospital, which occupies a commanding position at the side of a main road running along the brow of a plateau on the outskirts of the town. Its buildings extend down the steep declivity for eighty or a hundred feet to a gentler slope, ending in a meadow. A very ample water-way leads, from the canalised river on the plateau, down the side of the hill, and supplies a concreted bath or tank about forty feet long, over which a thatched shed is built. This is the bathing and clothes-washing pool for the patients; and an excellent and much-appreciated arrangement it is, the fresh, clean water always running through it in lavish abundance. Not far off is the kitchen—a rustic structure composed of teak frames panelled with plaited bamboo and roofed with atap, a capital style of thatch made of the leaves of the nipa palm, which is much in vogue in Java.

### The Wards.

Hard by, where the ground begins to slope less steeply, are the wards; and these are constructed entirely of bamboo, with the exception of the tiles with which they are roofed. In the ground bamboo would very soon decay, of course, but the climate of Buitenzorg is so free from strong winds that it is found sufficient to stand the corner posts and other bamboo uprights of the frame on an even-topped stone without any fixing whatever. There runs down the centre of each ward a roomy platform, also constructed of bamboo frames and bamboo plait, upon which the patients recline, and which does duty for a bed-place at night. The roof is ingeniously stayed to the centre of this by bamboos. A similar platform runs along either side of the ward, leaving an aisle or gangway space about four feet wide between it and the central platform. The entire ward is floored with concrete.

### The Patients.

Most of the patients at the time of my visit were Chinese; and there were about six hundred of them. They were not very bad cases on the whole, and the Javanese doctor had some little trouble to find one with the typical beri beri gait to show me. The medical superintendent informed me that the patients come from all parts of the Dutch East Indies to this hospital, but principally from Sumatra; and that the change of residence from the locality in which they became affected with beri beri to a strange place—in this instance to Buitenzorg—forms one of the most valuable features in the cure. He stated that he adopts no specific drug treatment, attending merely to symptoms as they arise; but that he pays attention to the diet on the lines suggested by Dr. Vordermann. Dr. Vordermann concluded that in countries where the disease is endemic persons dieted on the decorticated white rice of Java, and not adequately supplied with nitrogenous food from animal sources, develop beri beri; while those fed on the red rice of the country remain free from it. Many, however, of the natives object to the red rice on account of its appearance; and to these the cortex is given separately in this hospital mixed into a rather nasty looking mess of potage, with native-made sugar and flavouring materials. They also receive an allowance of meat, dried fish, eggs, and vegetables, arranged regularly on alternate days in the week, and distributed over three meals daily.

### Nursing by Malays.

The nursing is done by two Malay attendants, and two Javanese qualified house physicians look after the patients in the absence of the medical superintendent, who visits the hospital every day.

### The Death Rate.

The treatment at this hospital appears at any rate to be successful in an encouraging degree, and the annual number of deaths during the last two years has only been twenty-four over a daily average strength of between six and seven hundred patients.

I left the Ruma Sakit Beri beri feeling much interest in what I had seen and heard there, and in the material it afforded me for comparison with what I knew of beri beri elsewhere. After a further little linguistic flutter with my obliging mentor and cicerone, he furnished me with his card for the directeur of the Hospital for Insane, Dr. Hofman; and I once more transferred myself into the ever-present "sado" for the drive to that magnificent asylum.

### Some of My Experiences as a Dispenser.

By Constance Bradbury, Resident Dispenser at Ryde Dispensary.

When I first began to learn dispensing, about seven years ago, very few women had taken up the work, but now it is a well-known and favourite profession, due no doubt to the independent life it offers, combined frequently with short hours. Still, like everything else, it has its drawbacks. It is essentially a lonely life, as it is not at all advisable to have companions about you when at work. Also it is a life of grave responsibility, and not, as a lady once suggested to me, merely "a pleasant pastime." There must be no wool-gathering and no castle-building mixed in gratuitously with the drugs.

In large hospitals the hours are often long, and the strain in such cases very considerable, especially when there is also a large out-patient department. If the dispensing of such institutions is undertaken by a woman she must be strong, and it is a great help if the committee will allow her to take a pupil assistant who has had a little experience, but would gladly give her services for a few months' further training of this description. I consider that the standing in a dispensary, combined with such close attention to detail, is far more tiring than the constant running to and fro in a hospital ward, and I have had experience of both.

### The Work at Ryde.

My work, however, in the Ryde Dispensary is anything but hard, and if it were not for my pupils I should have a great deal of spare time on my hands. When first I came here my patients had never heard of a lady dispenser, and, I fancy, somewhat doubted the efficacy of medicines mixed only by a woman's hand; but after a time, like the barbarians at Melita, when they "saw no harm come they changed their minds;" and I was intensely amused one day when a small child returned with her bottle, being sent back by her mother to ask if *I* thought that what the doctor had given her was the right sort of medicine! It is remarkable to find how the poorer patients prefer a thick, dark mixture to one which, if it happens to be colourless, they conclude is only water, and consequently often invite their fellow sufferers to take a "sip" while waiting their turn. I suppose most nurses have heard the story of the widow woman who, after watching the chemist carefully weigh out a grain of calomel and then divide it into two powders, exclaimed: "There now, you needn't be so awful stingy, seein' it's only for a poor faitherless bairn." I wonder how they would like it if we were more liberal with poisonous drugs. A single grain of many powerful alkaloids would be enough to cause a child's death.

### The Responsibilities of Dispensing.

I think that girls very often forget the serious responsibility they undertake when they decide to become dispensers. They seem to think a couple of months' practice is all that is required; in fact, when I began the work myself it was rather with this impression. I had been a nurse, and had, unfortunately, contracted typhoid fever, which prevented my going on with nursing for a time. In the meanwhile I attended some chemistry lectures, with no definite object in view, but I found them so interesting that later I did some practical work in the Newnham College Laboratory. Having gained a sufficient knowledge for my purpose, I then went in for a course of practical dispensing at the Leamington Hospital under Miss Swain, and it was while there that I decided to give up further thoughts of nursing and stick to my bottles. We had such numbers of out-patients almost every day that the practice was unlimited, but the hours were very long, and we often had no break from mid-day until late in the evening, tea on busy days being an impossibility. When I had finished my course there I obtained an appointment at a small hospital in North Wales, giving my services in exchange for board and lodging. The work there was a mere nothing to what I had been accustomed to, but it was good experience for me to begin on my own account in a small way where there was no hurry and no "impatient out-patients" grumbling at the time they had been kept waiting.

### Miss Bradbury's Pupils.

Since then several of my pupils, after passing the assistants' examination of the Apothecaries' Society, have done the same for a few months, and have afterwards obtained good appointments with salaries on their own account. Others have gone straight from me to a post where they have immediately received remuneration for their work; one, indeed, the day following her examination. I get such numbers of letters asking for particulars about the examinations, salaries, and chances of work that if I were to answer them all "with full details," as requested, I should have to engage a private secretary. Some of the letters are certainly amusing. One was from an anxious relative who asked me if I would kindly write and persuade her daughter to give up the idea of being a nurse, as "having failed myself," she thought I might use my influence in this direction. Another fond parent, after telling me of serious family troubles, wanted me to teach her daughter dispensing (for nothing), as the girl wished to enter the medical profession, and she thought she might manage to do so through "the back door of dispensing."

### A Field for Women.

There is certainly a large field open for women as dispensers, and from a varied experience I can confidently say that, if they are suited to the work, employment can be found, especially with the minor qualification of the Pharmaceutical Society. They must, however, first get practical experience in a hospital or a dispensary, and a good grounding in the theoretical work as well. Examination work and the actual dispensing of medicines for patients are two different things; both are necessary, and it is a somewhat difficult thing to decide how women can get the two to the best advantage. In the case of youths it is different. They naturally apprentice themselves to a good chemist, and at the end of their time spend some months in one of the many schools of pharmacy in London, where they work up for their examinations before finally entering business on their own account. I think, however, it will be a long time before women seriously compete with them in this line, or at any rate the class of women for whom these few words are written. There must always be a number of small hospitals, where gentlewomen will prefer to work for a lower salary rather than enter into a business in which they must give up much that they hold dear; and because women will work for charitable institutions at a lower wage than men, it does not follow—as is sometimes suggested—that the work is less well done. A man has to earn enough to support a wife and family, and would often not consider it worth his while to dispense for a small institution, whereas a woman having, as a rule, only herself to think of, is glad to be able to keep herself, and at the same to curtail in any way the expense of those charities by which the poor benefit so largely. I feel sure if more hospitals would employ women as dispensers they would find the plan both economical and satisfactory.

### For Mental Nurses.

We are asked to call the attention of mental nurses to the approaching examination for the certificate in nursing and attending the insane to be held by the Medico-Psychological Association on Monday, November 5th. No names can be received after October 8th, and all information will be supplied by the Registrar, Dr. Benham, City Asylum, Fishponds, Bristol.

### Echoes from the Outside World.

### AN OPEN LETTER TO A HOSPITAL NURSE.

WAR as ordinarily understood has now ceased in South Africa. The proclamation by Lord Roberts announcing that the Transvaal will henceforth be an integral part of her Majesty's dominions means that the whole of the territory over which Ex-Presidents Kruger and Steyn formerly exercised authority has been formally passed into the possession of Great Britain. Henceforth those who resist British rule must be regarded simply as rebels against the Queen's government, and they will, of course, be treated as such. It does not follow that there will be an immediate termination of the campaign, which, in fact, cannot end until the rebels cease to worry and our weary soldiers are at rest. The moral influence of the proclamation can hardly fail to be considerable. It will destroy the last lingering hope of any sanguine or confiding burghers who have hitherto imagined that their leaders would not yield to force which, in this case, has proved a very sufficient remedy.

THERE have always been people who have maintained that Mrs. Maybrick was wrongly convicted, and such persons naturally cite with much satisfaction the letter which has just been published from the late Lord Chief Justice, written so far back as June 27th, 1895, expressing himself to the same effect. There is certainly no possibility of doubting the meaning of the communication from Lord Russell of Killowen, but on the other hand, it is no news to learn that he never believed the unfortunate woman whom he defended with such consummate ability to be guilty of the murder of her husband. It must also be remembered that in spite of the eloquence of her counsel—an eloquence which probably was additionally impressive because the advocate felt his cause was just—the jury refused to acquit Mrs. Maybrick. To argue that England cannot detain in prison anyone whom a particular Lord Chief Justice declares ought never to have been convicted is to suggest judicial infallibility. Moreover, the present Home Secretary has long since fully considered the representations made to him by Lord Russell, and one may be quite sure that he would not have disregarded them unless he had come to the conclusion that they were not irresistible.

WE have been much interested in the lace making in the neighbourhood of Honiton. I learn that for a long time the trade has languished, and that those Devonians who loved their county and were proud of the beautiful lace it once produced were sad accordingly, and looked back with regret to the days when the Queen's wedding veil had been made at Beer, and when no bride would have thought her trousseau complete without some costly examples of Honiton lace. But lately several patriotic ladies have been interesting themselves in the industry, and in organising regular bands of workers in various villages in and around Honiton, with a result that a revival is taking place, and it is becoming quite a common thing to see the women sitting at their cottage doors in the evening busy with their cushions and bobbins, or, as they call them in untechnical parlance, "sticks." A great deal of the work they do is effective enough, even if coarse in pattern, but being moderate in price it finds a ready market, though by no means of the delicate workmanship for which Honiton lace was once famous. But some of the women, on the contrary, have attained a high degree of excellence, and produce some lovely sprays. As a rule, the cottagers do not make up the flowers, &c., into collars, ends, or ties themselves, but sell them direct to a lady who mounts them and sends them to London and elsewhere for sale. Most of the women seem only to work at their pillows at odd times, in the same way as cottagers do elsewhere at knitting or crochet, and there is one advantage in lace making over other needlework for a busy housewife, namely, that as the fingers never touch the cotton, it is a matter of small moment if the hands are rough and not exceedingly clean. Possibly, the workers might regard it as a source of income, rather than as an addition, if it were better paid; but a rose and short stalk, taking three-quarters of an hour to make, is valued one halfpenny when finished, or, to encourage systematic continuance, sevenpence a dozen, when the whole number is sent in complete to head quarters at once. It is rather nice to be able to buy the "motifs" separately, as they lend themselves to dress decoration in so many ways impossible when the lace is already mounted into some set form. Nearly every village of any importance has a sort of little depôt where lace can be bought, and in the larger towns the lace shop is quite a feature.

SIDMOUTH is far better known than Budleigh Salterton, but in spite of the fact that it has more shops and more visitors, it is still impossible to call it "a fashionable seaside resort"—to my mind a most objectionable commendation. It is true that a great many of the lovely houses, and even mansions, on the hills around are occupied by titled folks and good old families, and that the general summer holiday-makers are of a very nice class; but the place itself is still quiet and the surroundings peaceful, in spite of a band, a parade, and a recreation ground. The situation of the town is very pretty, nestling down between the hills, with the sea in front, and the trees and fields stretching far away in the valley behind, and though to my mind an ideal spot for spring or autumn rather than summer, I am told by some friends who are stopping there, they have never once felt it oppressively hot, even during the warmest weather. The shops are good—notably several first-rate fruit shops, where the plums, peaches, and nectarines are splendid just now—and there are several places of interest in the neighbourhood, particularly the house where the Queen lived with her parents in her early childhood, and where her father died in 1820. A west window in one of the churches, St. Giles, was given by Her Majesty in memory of the Duke of Kent.

THE walks around Sidmouth are glorious. Of course, you cannot stir outside the town without mounting a hill, but if you object to travel on Shanks' pony you can do so behind someone else's steed, and the panorama opened before you when you reach the top is enough to repay any extra exertion. The view from Salcombe Hill, east of the town, is said to be one of the finest in Devonshire, and that from High Peak Hill on the west side is almost as beautiful. There is one particularly delightful trip, which need not be fatiguing if it is a calm day, at least for those who like to be on the sea. It is only a short row from Sidmouth to Ladram Bay, a sheltered beach surrounded by red sandstone cliffs—a distinctive mark of this part of the country—which the waves have chafed and worn into quaint shapes and jutting headlands, with here and there natural arches and caves. The approach to the beach from the land is down a little ravine, and sometimes there is no one there at all, nor a sound to be heard except the wash of the water and the cry of the seagulls. Occasionally a party of bathers arrive with a tent, for it is a capital place for a dip, there being neither treacherous pools nor gulfs to fear. The return journey for the tourist from Sidmouth should be made by land. The ravine leads into a typical Devonshire lane, with hedges on each side ten to twelve feet high, covered with ferns and flowers, nut trees, and blackberry bushes. Winding upwards on to more level land, the road ultimately terminates in a wood more than half a mile through. As you emerge the scene before you beggars description. Before you on one side is the sea and the rugged cliffs, on the other the hills and valleys, woods and streams, so typical of Devonshire, and in front heather-clad hills gay with purple blossoms and waving green ferns. The descent into Sidmouth is, in places, very sharp, though not particularly sudden. The walk by the side of the cliffs is most picturesque, but there is also a very good road.

### Notes on Hygiene.

### COLD BATH-ROOMS.

By a Correspondent.

It cannot be said that the average bath-room deserves to be added to the list of those "home comforts" which are the boast of British householders. Even in the cosiest homes the bath-room is apt to be the one fireless, cheerless spot, and doubtless to this circumstance a large percentage of untraced colds and chills are due. We are a comfort-loving people, but we take our comforts unevenly. Many persons who spend their "winter of discontent" in an easy chair in the drawing-room regard a bed-room fire as a reprehensible piece of "coddling." A warmed bath-room is considered by many such people as a still greater advance towards wilful luxury. The chilled bath-rooms wherein the majority of us indulge in our ablutions are the outcome of domestic prejudice similar to the superstition which bars fires after the first of May—"because the chimneys have been swept."

There may, of course, be a danger to health in putting too fine a point on comfort and ease. But the average Britisher—with his climate of multiple moods and quick changes—stands in little danger of becoming a "hot-house plant." For this is the terrible bogey held up by old-fashioned persons when a rising generation very sensibly asks, during bitter wintry weather, for a warmed bath-room. On a cold night, the mere thought of the average bath-room causes a shudder of chilliness. After spending the evening in a genial, cosy, after-dinner atmosphere, it needs some courage to take a bath. In nine cases out of ten the British bath-room is rigorously dedicated to the powers of frost and chills. A cold linoleum or oilcloth frigidly covers the floor. Sometimes the walls are tiled; if not, they are covered with an eminently sanitary, but uncommonly cold-looking, washable paper. The bath itself is necessarily made of material of sub-normal temperature. Grateless and stoveless, there is not a glimmer of heat or warmth to encourage those habits of cleanliness which are proverbially akin to godliness. A felt bath mat is the only warm, comfortable spot for the eye to rest upon in the icy oasis. At the boundary of the mat begins the frost-line of the oilcloth region.

The fully-dressed intending bather shivers on the threshold. To undress in such a North Pole chamber is a fearsome thought; but the ordeal has to be faced. The warm bather of the household has the better part, for he usually takes his tub at night, hurries over the process, and quickly seeks a warm refuge "between the blankets." Old people, however, run a serious risk in exchanging the warmth of an evening sitting-room for an "undress" condition in a chilly bath-room. Liver congestions, general chills, and bronchitis are the bogies which do literally haunt the unwarmed bath-room, especially during such cold spells as we have lately suffered from.

Many people who practise the salutary and hardy habit of a daily cold tub are under the impression that the temperature of the room in which this is taken is a matter of no importance. Now this is a great mistake. The benefit of the cold bath is reached by the first chill of the plunge resulting in a secondary reaction. If a cold tub be taken in an unwarmed bath-room the first "chill" is sure enough. But the reaction is a matter of doubt and uncertainty. On getting out of the bath the frozen atmosphere of the room may—unless the bather have a strong vitality and an admirable circulation—result in a second chill. In the face of the bath-room's bitter cold the bather gets up no reaction. Thus he not only loses the specialised benefit of the cold tub, but he runs the risk of a congestive cold.

It is a matter of common, everyday knowledge that many persons are able to advantageously take a cold bath if this be followed by a brisk rub down before the fire. Take away the fire and such bathers experience only the chilliness and depressing effect which must necessarily result from a cold bath not followed by reaction.

Congestions of lungs, liver, and intestines, and other internal chills are the logical sequelæ of cold bath-rooms. Of course, many persons escape the logical sequelæ of life; but the home hygienist is often able to trace in the unwarmed bath-room the origin and cause of those otherwise mysterious liver chills, congestive conditions and winter diarrhœas which are so prevalent during a cold snap.

The disciple of the cold bath, who has the Spartan courage to keep to his wholesome custom, even when the thermometer registers many degrees below the freezing point, believes that he is fulfilling all hygienic requirements when he acts up to the philosophy of friction, and rubs and scrubs his skin in an endeavour to assist reaction. He is quite right in his efforts. But the blood, thus encouraged to the surface, is apt to be driven back suddenly and sharply by the frigid atmosphere, which from time immemorial has been thought to fit bath-room requirements. There is little question that, in the house of the future, a fireplace or a regulation heating apparatus will be regarded as even more necessary to the bath than to the dining-room. But there is no time like the present, and we of the modern generation need not perform our matutinal wash in a congealing, blood-freezing temperature, just because the traditions of our grandparents as to the benefits of "hardening" constitutions by every conceivable manner of discomforts, die hard. This is the day of portable stoves and practical and inexpensive methods of heating by oil.

The housewife can rescue her bath-room from an ice-bound fetish by the use, in severe weather, of a small oil stove at three shillings and sixpence. Or she may rise to the superior heights of a thirty shilling bath-room lamp stove. In either case, generations of the bathers of her household will rise up and call her blessed. At any rate, they will do so when the snow lies thick on the bath-room window-sill, and the water pipes threaten to freeze at the main.

### Presentations.

West London Hospital.—The board of management of the West London Hospital have presented Sister Thomas, better known at the hospital as "Sister Accident," with an illuminated testimonial and a purse containing £16 16s. The medical staffs, past and present, have also presented her with a purse containing £43 and an illuminated certificate. The address prepared by the board of management runs as follows: "The board of management having before them at their meeting, held 9th April, a letter from Sister Mary Thomas, in which she resigns her appointment, and it appearing that on the 28th May next she will complete 26 years' service, it was resolved, 'That the board hear with regret of the resignation of Sister Thomas, thank her for her faithful service, and express their best wishes for her in the future.'" This resolution was subsequently engrossed and sealed with the common seal of the corporation, and presented to Sister Thomas, the seal being affixed at a meeting of the board last month. Sister Mary Thomas was trained at St. Bartholomew's, and remained at that hospital for the first seven years of her nursing career. She was then appointed sister of the accident ward at the West London, and continued in charge of the ward after the new wing was opened, although it is no longer used for accidents. In her matron's opinion "she is all that a nurse ought to be." She is still active and strong, and has no thought of leaving the profession. After a holiday she proposes to take up private work.

### Notes on Rectal Nursing.

BY A TRAINED NURSE.

### II.—OPERATIONS.

If an operation for hæmorrhoids is decided upon, the patient is commonly prepared in the following manner: A dose of castor oil will be ordered two nights before the operation, if this is fixed to take place in one of the early morning hours. The night before the operation the patient is given a full hot bath, and the anus is well washed with soap and water. On operation morning a cup of tea will be allowed at about seven a.m., supposing the operation to be fixed for nine or half-past nine. A pint and a-half injection of warm barley water or very thin gruel should be given two hours before operation, and the nurse should roughly measure the result so as to be sure that all the fluid injected has been returned.

The dressings and requirements for operation differ so widely with the individual surgeon that these cannot well be specified. Usually flannel T bandages are employed, and iodoform or blue cotton wool forms the dressing. A small pad smeared with eucalyptus ointment or lint saturated with carbolic oil is placed over the ligated piles with a large external pad of blue or absorbent cotton, which is kept in place by a flannel T binder.

The day after the operation the external pad will be changed and the surrounding parts bathed, but the wound dressing will not usually be touched till the second morning. It will then be loosened by syringing, and the part will be cleansed night and morning with hot soap and water. Boracic or iodoform powder is frequently used to sprinkle the parts, and over this a light lint dressing smeared with eucalyptus vaseline.

The diet should consist of arrowroot, milk, patent unmalted foods, chicken and mutton broths. On the third day a little white fish may be given, but very little solid food or food leaving fæcal residue is suitable, as the bowels must not act till the sixth day after operation. If much solid be allowed the patient will suffer considerably from flatus and general intestinal fulness and discomfort. For some hours after the operation the nurse must look at the dressings periodically to see whether hæmorrhage has set in. If too much blood appears on the anal pad, the nurse must summon the surgeon. Meanwhile she can exercise pressure on the pad and tighten the T bandage. If this does not materially decrease the bleeding, she should syringe the rectum with ice-cold water. The patient must be placed on the left side with a pillow under the pelvis, and the part covered only with a thin towel so as to keep it as cool as possible. In preparing for a hæmorrhoidal operation the nurse should make ready all the appliances that are needed for plugging the rectum. In case of hæmorrhage after operation the surgeon may have to resort to plugging, and everything should be ready for the emergency. A conical sponge, about the size of an egg, is needed. The nurse can easily cut out an ordinary small sponge to this shape. A stout tape should be tied firmly round the centre of the sponge. Wrung out as dry as possible from the same antiseptic solution as was used at the operation, the sponge is pushed some three to four inches up the rectum, the tape ends being left outside. The remaining rectal space is plugged tightly with cotton wool wrung out in a sub-sulphate of iron solution, or the sub-sulphate in powder is sprinkled dry on the cotton wool. If the patient be suffering from much flatus the surgeon may direct that a hollow rectal tube be placed inside the sponge, so as to allow of its free passage. The tapes hanging from the sponge are tied across, and thus the plugging is tightened.

For the first few days the female patient should pass the water by means of a small kidney-shaped bowl whilst lying on the face; but if not able to manage in this position she may be allowed to kneel. This prevents the strain on the rectum which is inevitably caused by the use of an ordinary bed pan. Catheterisation may be necessary, but the hot sponge over the pubes should always be tried before the catheter is resorted to. Secondary hæmorrhage may occur between the fourth and seventh days. In a normal case a dose of castor-oil will be given on the sixth morning after operation. When the patient feels that the medicine will shortly act a 3 oz. olive oil injection should be given, which will materially soothe the great pain experienced when the first motion is passed. The patient must not be allowed to help herself with the bed pan for the first few days, and the nurse must carefully wash the parts with hot soap and water after every movement. Each stool must be examined for blood, and traces of this will almost invariably be present for the first few days. A small anal ulcer may follow the operation for hæmorrhoids; that is rare, but possible. On account of the tenderness and sensitiveness of the parts the enema syringe needs to be used with gentleness and care. Any roughness may cause an abrasion which may readily pass to fissure or ulceration. In a long standing case of piles such sequelæ are easily set up, owing to the unhealthiness and possible ill-nourishment of the mucous membrane of the rectum and anus.

### Nursing in a Colliery District.

BY A DISTRICT NURSE.

THE life of a district nurse in a brand new district is not all honey, as some people think, though it is deeply interesting, and also has its humorous side. It requires a deal of tact and patience, a power of holding one's own, together with the hide of a rhinoceros. I came into this colliery district just over a year ago. The first few months seemed like one long dark night, I thought the dawn would never break (it is not mid-day yet). On looking back I quite think the people had, from their point of view, as much difficulty with me as I had with them. They quite expected to find the nurse a great help to them, but in their own way she was to carry out their instructions, even in the way of making beds and poultices. "I have made poultices these forty years, but never saw any done that way before," said an old lady of sixty. That nurse had come to help them by teaching them a better way of doing things was quite a new idea, and did not take at all at first.

### THE "FOINE" TALK DIFFICULTY.

One of our difficulties was the people could not understand what they called my "foine talk," I could not understand what they termed their "pit or rough talk." Once I asked for a mug of water. After some considerable time up came a woman carrying a large bread-pan nearly full asking if it was enough. How we laughed. Had I asked for a cup of water she would have understood.

### PATIENTS HARD TO MANAGE.

After explaining, visit after visit, the reason why typhoids might not sit up or get out of bed, why they must have drinks at stated intervals, and not just when the friends thought of it, or on the rare occasions when the patients asked for it, I would return to find the patients had been in and out of bed at their own sweet will, and nourishment had scarcely touched their lips. Weeks later I found that, though I had been listened to patiently, the friends had either not understood a word I said, or when they had understood it was all too new for them to grasp. One old lady on parish pay, suffering from paralysis, talked incessantly while I attended her. I kept asking the daughter what she wanted, and was usually told a drink; the drink was given, but the talking never ceased. She was swearing at me for what I was doing, but I never knew till I was told months after. Washing and changing was, of course, a great difficulty; and in the words of Chevalier, "peculiar, most peculiar" were the ways employed to prevent my doing either.

No Hot Water.

Once there was no hot water, not even in the boiler, because it was Friday; on Fridays the kitchen fire was always let out for cleaning purposes. I politely asked for a kettle, and boiled the water in the patient's room, not knowing this was a ruse to prevent my bathing those poor rheumatic joints. Often I was told there were no clean clothes for the patient. My astonishment usually brought forth the information that they were washed but not aired. So meekly I used to arrange the clothes in front of the fire for half an hour or more, not so much to air them, for they seldom needed it, but to get the people's confidence, and to teach them that what I was doing was really necessary for the sick person's comfort. When no change of clothes were there, sometimes the relatives would relent and promise me them by the next visit, but it was never for the good of the patient—always to please me. Some said of me, "Thou is a queer woman; we never seed anyone like her afore. Why you never sees her in a temper; she's always the same, she is."

Nursing for Pleasure.

Of course they had never been accustomed to having an educated woman amongst them, practically no district visiting being done here by those the people call the "better end" or those "up sticks," the former being people of birth, the latter those who have risen. Now a very great deal of all this is changed. I can't call often enough to please some of them, and when they expect me they have everything ready for me, and will send into the next street to borrow a night dress if they think I will want it. They all seem to believe I nurse them not because I am earning my living, nor because they are reaping great benefit from my attentions, but because doing so is the only pleasure I have in the world!

A Little Convalescent.

To a very funny little boy convalescing from pneumonia I gave a toy pipe. He did not seem to know what to do with it, so I put it in his mouth and left him. He held it there till his mother took it from him, thinking he was getting tired, when a small brother, aged five, said, "Thou mon tak that out o' thee mouth when thou woman put it in, thou 'ilt die." Perhaps they thought the pipe had some connection with the thermometer. The children are wonderfully easy to manage, possibly because the mothers threaten them with nurse when they are naughty, such as "Hold thy din or thou nurse will be coming."

### TRAVEL NOTES AND QUERIES.

Rules in Regard to Correspondence for this Section.—All questioners must use a pseudonym for publication, but the communication must also bear the writer's own name and address as well, which will be regarded as confidential. All such communications to be addressed "Travel Editor, 'Nursing Mirror,' 28, Southampton Street, Strand." No charge will be made for inserting and answering questions in the inquiry column, and all will be answered in rotation as space permits. If an answer by letter is required, a stamped and addressed envelope must be enclosed, together with 2s. 6d., which fee will be devoted to the objects of the "'Hospital' Convalescent Fund." Any inquiries reaching the office after Monday cannot be answered in "The Mirror" of the current week.

Nursing Institutes on the Continent (A. C.)—You have given no pseudonym, so I must use your initials. At Alassio, on the Riviera, in the Gulf of Genoa; address Lady Superintendent, Chalet Santa Croce. At Nice, the Hollond Nursing Institute, Villa Soleil, Avenue Thiers; at Cannes, a branch of the last-named at Rue Montaigno, Bd. Foncière; at Hyères, a similar branch at 60, Avenue Gambetta. At Mentone there is an English nursing institute of which I know nothing, but it is probably quite reliable. There is also one at San Remo, 18, Via Roma. In Southern France I know of no institutes other than those on the Riviera.

Engadine (Sister Norah).—Your desire for secresy makes it very difficult to me to answer. The place you mention is very expensive for those staying there. I do not think it would be possible to live there for less than 6s. 6d. per day, and to manage even that you must be an experienced continental traveller. I do not think that you could rely upon work; most invalids take their nurses with them. The cost of journey second-class is £4 7s. 9d. It would be no cheaper to go by sea to Genoa, but dearer. It is a very risky thing for a nurse to try work on her own account on the Con tinent, and I should strongly advise you not to try it.

### Everybody's Opinion.

[Correspondence on all subjects is invited, but we cannot in any way be responsible for the opinions expressed by our correspondents. No communication can be entertained if the name and address of the correspondent is not given, as a guarantee of good faith but not necessarily for publication, or unless one side of the paper only is written on.]

### A DANGEROUS PRACTICE.

"An Old Hospital Matron" writes: The highly-dangerous practice of using heat to melt beeswax and turpentine for floor-polishing is quite unnecessary. If the turpentine is poured over the wax and allowed to stand all night it will be ready for use in the morning, and polish much better than if melted by heat. The death of Florence Plush, of Charing Cross Hospital, owing to fire from the habit of melting the polish on the stove will, I trust, be a warning to ward maids and scrubbers employed in institutions.

"Matron" writes: I see in The Hospital of August 25th an account of a sad death from burning due to the upsetting of beeswax and turpentine set to melt on a stove at Charing Cross Hospital. Since beeswax and turpentine are in such constant use in polishing floors, I think it cannot be made too widely known that there is no need to place them near fire or heat of any kind. If the beeswax is cut fairly fine and covered with turpentine it will be quite dissolved in twenty-four hours without any heat. After having a slight fire here several years ago from ignition of the mixtures I have never since allowed it to be placed near fire, and have found no difficulty in getting beeswax dissolved.

### A PRIVATE NURSING INSTITUTE.

"Disgusted" writes: I answered an advertisement in The Hospital for June 30th for a post on a private nursing staff. I had an interview in town, and was engaged to join on July 30th. I asked to see the head of the institute, and was told she was just now out of town. Now, as a matter of fact, she does not live there. The institute is left to an untrained woman. Then, again, my first case was a short one, and when I returned to the institute I was sent with others fourteen miles out of town to a Young Women's Christian Association. As the institute was full there was no accommodation for me. Surely it is the duty of the person who engages the nurses to inform them of these arrangements.

### NURSING FEVER IN AMERICA AND ENGLAND.

"M. C. N." writes: Some few days ago I met an American lady who has been for many years matron of one of the best hospitals in her own country. She had heard that I was especially interested in fever work, so she began to talk on that subject at once. She said that in New York there were no isolation hospitals. If fever breaks out in a block it is notified, and the city authorities cause a sign to be put on the door. No one is allowed to go near the family, nor are the relatives of the afflicted one allowed intercourse with the outside world until the infectious stage is over, when the city authorities again appear to fumigate. My friend told me of one poor woman, who with her three children had nowhere to go, and spent the day in the park whilst this very necessary performance was proceeding. Now, in contrast to this, I should just like to tell the readers of your valuable paper how these affairs are managed in a seaside town not an hour's run from London. There is a sanatorium belonging to the corporation, with a little gentlewoman at the head as matron. A woman in every sense of the word, true and just in all her dealings, and who has the gift of winning the love and respect of her nurses, making them feel they would work "the nails off their fingers" to save her trouble or annoyance. Hers is no easy post, yet she does her duty evenly and faithfully, and to her is due the credit of the sanatorium being the popular place it is to-day. There is a medical officer of health, but any doctor in the town may send in his cases and "follow them up." Forty-eight beds is now the limit, but the Corporation has already planned further extensions. So anxious now are people to get their relations admitted that often there are no empty beds. The hospital consists of three blocks—for enteric, diphtheria, and scarlet fever. They are surrounded by ample grounds, where, weather permitting, the convalescents can wander about, and where swings, cricket, football, &c., are provided for those who are able to enter into such sports. Most people have a feeling of dread upon seeing a "fever van," but here again is another "up-to date" arrangement. The sanatorium ambulances look like an extra roomy brougham, well swung, and with pneumatic tyres. The interior is beautifully fitted up with every modern improvement. When going for a "case" a nurse and sanitary inspector always accompany the ambulance. The patients are "all sorts and conditions," rich and poor, high and low. There are seven permanent nurses on the staff, and in the autumn extra. Each block has its own wardmaid, who does the scrubbing and general cleaning, and the bath-rooms, lavatories, and linen cupboards are fit always for inspection. As for the cleanliness of the patients, all I can say is that some of them while with us have had more baths than they ever had, or will have, during their lifetime. Ours is at times (especially in enteric) sadly anxious work, but we always know we have matron's sympathy and the kindly courtesy and encouragement of the doctors. It is understood that where possible patients are removed from their homes at night, so as to avoid notice, and very early the next morning our men go to fumigate the room and bring away all clothing, &c., to be disinfected by means of the Washington Lyons. All this facilitates matters for the patient's friends, enabling them to continue their usual duties, happy in the thought that their dear one is having every care and attention.

### THE UP-COUNTRY NURSING ASSOCIATION.

"A Nurse in India" writes: It may interest nurses contemplating coming out to India to know something of the life they are likely to lead should they be persuaded to sever their connection at home to work in this country. I remember reading some years ago a letter in The Hospital "Nursing Mirror" about the Up-Country Nursing Association. Among other things the writer said the nurses expect to be invited to every fashionable function of the season, or words to that effect. I thought no more about this letter till "Kismet" brought me out, and here I am working under the auspices of the ladies of the committee. Often, when at home, I heard people speak in high praise of a nurse's life out here—how much was thought of her work, how kindly treated she is by the wives of the leading civilians—and I formed the opinion that India was a veritable paradise for a nurse; so that I was glad enough to get an opportunity of seeing the country for myself. Before leaving home I had grand ideas of helping the poor native women. I had read so much about them, and thought I should be able to do something towards alleviating their suffering; but I was not long in the country before I discovered that, even in a small way, my services would not be needed or asked for in this line. Our head-quarters are in a lovely hill station. The scenery is superb, and the climate, except during the rains, perfect. Our work extends to the whole of the North-West Province and Oudh. We sometimes spend months together at a case, on the hot and steamy plains in summer—not that we ever think of complaining. We are here for a few days between cases, and when this happens in summer how thankful we are that the "Powers" saw fit to select this lovely settlement for our head-quarters. Indirectly, the association is under the management of a committee of ladies, who, in my humble judgment, know very little about nurses or nursing; if they did they would take more interest in the association generally, and in the workers personally. Directly, the management is in the hands of the honorary secretary, one of the ladies of the committee. Our honorary secretary is frequently changed, which is a very great pity. The last lady was just beginning to understand our aims and aspirations, and to take an interest in us personally, when she was called home. When the association was first organised a nurse was addressed by the ladies, the honorary secretary, and patients alike as "Sister," and letters to her were addressed "Miss," or "Sister So-and-So." This courtesy is now deemed unnecessary; she is addressed "Nurse," and if there is occasion to write to her the envelope is addressed "Nurse So-and-So." When it is taken into consideration that the Home Committee take pains to procure intelligent and educated women to fill vacancies, it will be easier to understand that not to treat nurses with ordinary courtesy is, to say the least, harmful to the interests of the association. For fear we should succumb to *ennui*, the committee very graciously subscribed to the station library for us, and we are now allowed a book or two to help pass a pleasant hour or so when off duty. I had a most laughable experience with the Babu assistant librarian the other day. In the morning I called to change a book. I was asked my name by the Babu. I told him it was "Miss ——." "We have no one of that name on the subscribers' list," said the Babu. I then told him that I belonged to the association, and we settled matters amicably when I pointed out we had been subscribed for collectively as "nurses." The home is conveniently situated, and the part allotted to the nurses is roomy and, on the whole, comfortable. There is a drawing-room, a dining-room, and each nurse has her own bedroom, which we try to make as pretty and cosy as we can. The drawing-room, thanks to the kindness of the last hon. secretary, has nice curtains and mantel drapery; the chairs would be comfortable if the springs were refitted, and a little money spent on new upholstery. For the first few months I suffered from what in my country is called *heimweh*, but after a time I got over it. Nursing out here is really interesting work, and one makes a few friends. The patients are thoroughly appreciative, and they are so kind, doing all in their power to make our stay with them during their convalescence pleasant. Social pleasures consist chiefly in invitations to afternoon teas.

### Appointments.

Glasgow Fever Hospital, Ruchlil.—Miss Jemima Broom and Miss Maria Bissett have been appointed Assistant Matrons. Miss Broom was trained at the Western Infirmary, Glasgow, for three years, and was afterwards charge nurse at Burnhill Poorhouse, Glasgow, for five years. Miss Bissett has been trained at the Worcester General Infirmary and the Victoria Infirmary, Glasgow. She has since been district nurse at Sunderland and Portsmouth, and charge nurse at the Borough Hospital, Croydon, and the Borough Sanatorium, Sunderland.

### Minor Appointments.

The Suffolk General Hospital, Bury St. Edmunds.—Miss Elsie Wallworth has been appointed Night Sister at this institution. She was trained at the Dundee Royal Infirmary, and worked for eighteen months on the staff of the Sussex County Hospital, and as sister at the Royal Alexandra Hospital, Brighton, for two and a half years.

Glasgow Fever Hospital, Ruchill.—Miss Mary Masson has been appointed Home Sister. She was trained at Gray's Hospital, Elgin, N.B., for three years, and the Belvidere Fever Hospital, Glasgow, for two years. Subsequently, she has been charge nurse for four years at the Belvidere Fever Hospital.

Manchester Children's Hospital.—Miss Margaret Carruthers has been appointed Sister. She was trained at the Cumberland Infirmary, and afterwards had charge of the children's ward. For two years she has held the post of sister of the children's ward at the General Infirmary, Macclesfield.

West Ham Hospital, Stratford, E.—Miss Mary Carruthers has been appointed Sister. She was trained at the General Infirmary, Huddersfield, and has lately held a post at the General Infirmary, Macclesfield.

Isolation Hospital, Muswell Hill.—Miss Mary Daisy Fardell has been appointed Home Sister. She was trained at the Victoria Hospital and the Isolation Hospital, Muswell Hill, and has since been staff nurse at the latter institution.

### For Reading to the Sick.

GRANT peace on earth, and after we have striven, peace in Thy Heaven.—*P. Pusey.*

Well if we pray till Thou awake !
One word, one breath of Thee
Soft silence in the heart will make
Calm peace upon the sea. —*Keble.*

Only the waters which in perfect stillness lie,
Give back an undistorted image of the sky.
—*Trench.*

**Reading.**

Peace is that settled calm happiness which is more quiet and lasting than joy, more noble and worthy than pleasure. Search the heart in which true peace dwells, and you will find it reaching down to the centre of life itself; pleasure, pain, joy, sorrow, may come and go, but peace abides through all. —*Fosberry.*

Peace can only be found in lowliness, and that lowliness is only real so long as we suffer ourselves to be abased under God's hand, time after time, as He wills. The means which he uses are precisely those most suited to our case; contradictions and blame from others, and our own inward weakness. We must learn to bear one and the other, from without and from within.—*Fénélon.*

Impatience and fretting under trial does but increase our suffering, whereas meek submission sanctifies all suffering and fills the tortured heart with peace amid its anguish.—*Guilloré.*

Pain, which by nature leads us only to ourselves, carries on the Christian mind from the thought of self to the contemplation of Christ—His passion, His merits, and His pattern; and thence further to that united company of sufferers who follow Him. He is the great object of our faith, and while we gaze upon Him we learn to forget ourselves.—*Newman.*

God for His service needeth not proud work of human skill;
They please Him best who labour most to do in Peace His Will.
So let us strive to live ! and to our spirits will be given
Such wings as, when our Saviour calls, shall bear us up to heaven. —*Wordsworth.*

Remember how in the night storm on the sea, when the disciples' hearts failed them for fear of that dim mysterious Form which drew near, half hidden by the darkness, the voice of their Master spoke instant peace. "It is I, be not afraid." If you indeed know who it is that cometh to you upon the waves of these afflictions, amidst the darkness of this trial, you will not be dismayed.—*Fosberry.*

Oh, how do all the ills of life fade into nothing; how would its sadness become gladness, its thorns a crown, if we but see in them the eternal hand of God, moulding us by them for everlasting glory.—*Pusey.*

If in every cross, in every ache and pain, and in every sorrow we could look upon the bright side, and regard each as a love token from the Father's hand—then, instead of finding it a hard struggle to bear with patience any thwarting of our will, or ease, we should truly give thanks, blessing Him who thus endeavours to make us, through fellowship with His sufferings, likeminded with Himself.—*Hare.*

Blessed Healer ! all our burdens lighten,
Give us peace, Thine own sweet peace we pray,
Keep us near Thee till the morn shall brighten,
And all mists and shadows flee away.
—*Baynes.*

### Notes and Queries.

**THE Editor is always willing to answer in this column, without any fee, all reasonable questions, as soon as possible.**

**But the following rules must be carefully observed :—**

1. **Every communication must be accompanied by the name and address of the writer.**
2. **The question must always bear upon nursing, directly or indirectly.**

**If an answer is required by letter a fee of half-a-crown must be enclosed with the note containing the inquiry.**

*Training.*

(230) (*a*) I want to become a Queen's nurse, but cannot get my people's consent. How can I gain the necessary experience, and how much will it cost? What is the nearest hospital to Bridgewater at which probationers are trained, and are there any nursing lectures and ambulance classes which I could attend in that neighbourhood ? (*b*) It is against the rules for anyone excepting nurses to study THE HOSPITAL and "Nursing Mirror"?—*An Anxious Inquirer.*

(*a*) The only satisfactory way to become a nurse is to enter a general hospital for three years' training. If you will procure from the Scientific Press (price 2s.) a copy of "The Nursing Profession: How and Where to Train" you will find all your questions, excepting those relating to local classes, fully answered. And further, if you take the book to your parents, and explain that many excellent institutions will train you thoroughly at no cost, possibly they will grant you your wish. (*b*) What a question ! Anyone can study whatever she likes.

*The Mattei Treatment.*

(231) Can the editor give me any particulars of the Mattei treatment for cancer, and of the success with which it met?—*Importunate.*

The remedies, which could not be distinguished from plain water, failed to affect the course of the disease or its termination when submitted to a public test a few years ago.

*Incurables.*

(232) Can you tell me of an institution, near Wales preferred, where a deformed, helpless, but intelligent girl could be taken. Payment made. —*Mary.*

The "Englishwoman's Year Book" or "Burdett's Charities" have lists of such institutions, and you might possibly find exactly what you want in either of them.

*Training.*

(233) I am anxious to obtain a short hospital training to enable me to take up private nursing. I am thirty years of age, and have been maid-housekeeper to a lady for nine years; she died last September. Could you tell me which London hospital, having a private staff, would give me short training and a small salary?—*Anxious.*

No hospital will give a short training and a small salary, but many excellent ones are open to probationers of your age which will give you the three years' course and a fair salary from the first. See the "Nursing Profession: How and Where to Train," or apply to the matron of the Middlesex Hospital, or the Nursing Sisters of St. John the Divine, 24, Drayton Gardens, South Kensington. See also our advertisement columns.

*Dispenser.*

(234) Will you kindly inform me where I could learn dispensing and get a salary at the same time?—*Dispensing.*

See Miss Bradbury's article in this week's *Mirror.* She would doubtless give you further particulars.

*L.O.S.*

(235) I am going to a maternity hospital to be trained for the L.O.S. Can one prepare for it beforehand by reading or attending lectures?—*A. W.*

You might find it a help to attend the maternity courses of the Midwives' Institute, 12, Buckingham Street, Strand.

*Madeira.*

(236) Would you inform me if Madeira's climate is considered suitable for persons with chest troubles; also if there is work there for nurses with the L.O.S. certificate?—*H. B.*

Madeira was once the favourite resort of persons with pulmonary troubles at one time, but fashion has changed. Perhaps it will change back again. There are plenty of nurses on the island. Write to the Emigrants' Information Office, 31, Broadway, Westminster, for the latest returns of a nurse's prospects in any colony to which you think of going.

*Home for Aged Woman.*

(237) Can you tell me of any asylum where an old woman of 96, unhappy in her daughter's home, can be received? – *Chriss.*

We fear that so long as the mother has a daughter able to support her, nothing can be done in the matter except on payment.

**Standard Books of Reference.**

**"The Nursing Profession: How and Where to Train." 2s. net; post free, 2s. 4d.**
**"The Nurses' Dictionary of Medical Terms." 2s.**
**"Burdett's Series of Nursing Text-Books." 1s. each.**
**"A Handbook for Nurses." (Illustrated.) 5s.**
**"Nursing: Its Theory and Practice." New Edition. 3s. 6d.**
**"Helps in Sickness and to Health." Fifteenth Thousand. 5s.**
**"The Physiological Feeding of Infants." 1s.**
**"The Physiological Nursery Chart." 1s.**

**All these are published by THE SCIENTIFIC PRESS, Ltd., and may be obtained through any bookseller or direct from the publishers, 28 & 29, Southampton Street, London, W.C.**

